# Comparison of semiquantitative and differential time to positivity methods for the diagnosis of central line-associated bloodstream infections in an intensive care unit

**DOI:** 10.1099/acmi.0.000029

**Published:** 2019-06-05

**Authors:** Theodoros Karampatakis, Katerina Tsergouli, Ekaterini Karantani, Anna Diamantopoulou, Eleni Mouloudi, Emmanuel Roilides, Angeliki Karyoti

**Affiliations:** 1 Infectious Disease Unit, 3rd Department of Pediatrics, Medical Faculty, Aristotle University School of Health Sciences, Hippokration General Hospital, Thessaloniki, Greece; 2 Microbiology Department, Hippokration General Hospital, Thessaloniki, Greece; 3 Intensive Care Unit, Hippokration General Hospital, Thessaloniki, Greece; 4 Infection Control Committee, Hippokration General Hospital, Thessaloniki, Greece

**Keywords:** central line-associated bloodstream infections, semiquantitative method, differential time to positivity, intensive care

## Abstract

**Introduction:**

Central line-associated bloodstream infections (CLABSIs) adversely affect patients’ hospitalization.

**Aim:**

We compared semiquantitative roll plate (SQRP) and differential time to positivity (DTP) culture methods in diagnosing CLABSIs.

**Methodology:**

A retrospective study was conducted in an intensive care unit (ICU) from January 2013 to August 2014. All ICU patients with suspected CLABSIs were included. Blood cultures were taken, while central venous catheter (CVC) tips were cultured using the roll-tip method. DTP was considered positive if CVC lumen blood cultures became positive at least 2 h prior to concurrently drawn peripheral blood cultures with an identical micro-organism. SQRP method was considered positive when ≥15 c.f.u. of a micro-organism identical to that of blood cultures grew. Measures of diagnostic accuracy were calculated.

**Results:**

SQRP displayed high sensitivity (94.7 %), while DTP showed high specificity (82.5 %). SQRP combined with DTP displayed 100  % sensitivity and negative predictive value.

**Conclusion:**

SQRP and DTP methods should be evaluated in combination.

## Introduction

Central venous catheters (CVCs) are very helpful devices, widely used during hospitalization of critically ill patients [1]. Central line-associated bloodstream infections (CLABSIs) are important complications of their use [2]. According to the Centers for Disease Control and Prevention (CDC), CLABSI is defined as a primary laboratory-confirmed bloodstream infection (BSI) in a patient with a central line placed for >2 calendar days, occurring at the time of, or within 24 h prior to, the onset of symptoms, in cases where the cultured organism is not related to an infection from another site [3].

CLABSIs may have a crucial impact on mortality and cost of patients’ hospitalization [4, 5]. Therefore, the prevention of such infections is of great importance and requires the implementation of optimal practices [2]. CLABSIs frequently necessitate removal of the CVCs [6]. However, studies reveal that in up to 80 % of cases, the removed devices are not the source of the patients’ symptoms [7].

Conventional methods that contribute to the diagnosis of CLABSIs include CVC tip cultures using semiquantitative or quantitative methods [8], paired quantitative blood cultures taken through catheter lumen or peripherally and differential time to positivity (DTP) methods [9]. The aim of this study was to compare the semiquantitative roll plate (SQRP) and DTP methods for the diagnosis of CLABSIs in patients of an ICU.

## Methods

This retrospective cohort study was conducted in thenine-bed adult ICU of Hippokration General Hospital of Thessaloniki from January 2013 to August 2014. All ICU patients with suspected CLABSIs bearing a CVC were included. CLABSIs were defined according to the CDC criteria [3]. BSIs with data on CVC tip cultures, as well as CVC lumen and peripheral blood cultures were studied.

Blood cultures were taken in aerobic and anaerobic bottles containing 5 ml each and incubated in a BacT/Alert 3D system (Biomerieux, Marcy-l’Etoile, France), while CVC tips were cultured using the roll-tip method [10]. Identification and antimicrobial susceptibility testing was performed by a VITEK 2 automated system (Biomerieux, Marcy-l’Etoile, France). Interpretation results, expressed as sensitivity, intermediate sensitivity and resistance, were determined according to the 2014 Clinical and Laboratory Standards Institute breakpoints [11]. Micro-organisms were considered identical when they were of the same species and had the same antimicrobial susceptibility profile.

DTP was considered positive if CVC lumen blood cultures became positive at least 2 h prior to concurrently drawn peripheral blood cultures with an identical micro-organism, as previously described [12]. DTP was calculated using the BacT/Alert 3D system. The CVC tip was removed approximately 48 h before or after blood cultures were drawn. The SQRP method was considered positive when ≥15 c.f.u. of a micro-organism identical to that of blood cultures grew, as previously described [10]. Sensitivity, specificity, positive predictive value (PPV), negative predictive value (NPV), accuracy, positive likelihood ratio (LR+), negative likelihood ratio (LR-) and diagnostic odds ratio (DOR) were used as measures of diagnostic accuracy and were calculated as previously described [13], with an exact binomial 95 % confidence interval (CI).

Normality of continuous variables was evaluated using Kolmogorov–Smirnov and Shapiro–Wilk tests according to sample size. Differences in non-normally distributed variables were evaluated using Mann–Whitney U-test, while in normally distributed variables Student’s *t*-test was used. Categorical variables were compared using Pearson’s chi-square or Fisher’s exact test. The level of statistical significance was set at *P*<0.05. Statistical analysis was performed using the Statistical Package for Social Sciences (SPSS, 22nd version, IBM).

## Results

**Fig. 1.**
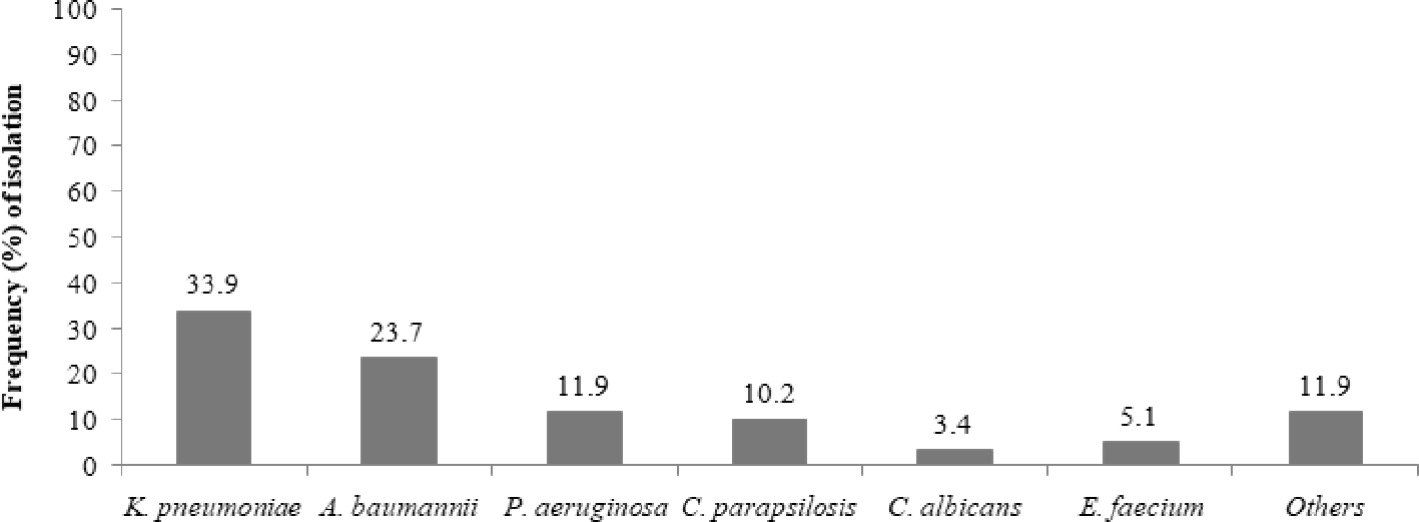
Frequencies of isolated pathogens in ICU patients with BSI. Others: single isolates of *C. tropicalis, E. cloacae, E. aerogenes, E. faecalis, S. marcescens, S. aureus and S. haemolyticus.*

A total of 59 BSIs were evaluated in 51 patients. The demographic data of patients are shown in Table 1. There was no significant association between CLABSI or non-CLABSI and patients’ survival. CLABSI was confirmed in 19 out of 59 BSIs (32.2 %). Overall, 27 out of 59 BSIs (45.8 %) were positive by the SQRP method, while 18 cases (30.5 %) were positive by the DTP method. Median time for DTP in these cases was 5.1 (IQR=20.8) h. *Klebsiella pneumoniae* was the most frequent pathogen isolated (33.9 %), followed by *Acinetobacter baumannii , Candida* spp. and *Pseudomonas aeruginosa* (23.7, 15.3 and 11.9 %, respectively) (Fig. 1).

**Table 1.** Demographic data of patients with CLABSIs and non-CLABSIs

Parameter	Total cohort	CLABSI	non-CLABSI	OR^*a*^ (95 % CI)	*P-*value
Patients, *n* (%)	51 (100)	19 (37.3)	32 (62.7)	–	–
Age (years), median (IQR)	56 (17)	56 (20)	55.5 (15)	–	0.60^*b*^
Sex male, *n* (%)	36 (70.6)	11 (57.9)	25 (78.1)	0.38 (0.112–1.327)	0.125^*c*^
ICU length of stay (days), mean (sd)	25.9 (15.9)	28.5 (20.3)	24.4 (12.8)	–	0.38^*d*^

*a*, Odds ratio.

*b*, Mann-Whitney U test.

*c*, Chi-square.

*d,* *t*-test.

For the diagnosis of CLABSIs, SQRP displayed high sensitivity (94.7 %), NPV (96.9 %), accuracy (83.1 %), LR+ (4.21) and low LR- (0.07). DTP showed high accuracy (74.6 %), high LR+ (3.31), low LR- (0.51) and higher specificity (82.5 %) than SQRP. SQRP combined with DTP presented the highest sensitivity (100 %), NPV (100 %) and DOR (79.4 %) (Table 2).

**Table 2.** Measures of diagnostic accuracy with 95 % CI

	SQRP	DTP	SQRP combined with DTP
**Sensitivity % (95 % CI**)	94.7 (71.9–99.7)	57.9 (33.9–78.9)	100.0 (79.1–100.0)
**Specificity %** (**95 % CI**)	77.5 (61.1–88.6)	82.5 (66.7–92.1)	67.5 (50.8–80.9)
**Positive predictive value (PPV) %** (**95 % CI**)	66.7 (46.0–82.8)	61.1 (36.1–81.7)	59.3 (40.8–67.1)
**Negative predictive value (NPV) %** (**95 % CI**)	96.9 (82.0–99.8)	80.5 (64.6–90.6)	100.0 (84.5–100.0)
**Accuracy %** (**95 % CI**)	83.1 (70.9–86.3)	74.6 (61.7–85.1)	77.9 (66.5–77.9)
**Positive likelihood ratio (LR+**) (**95 % CI**)	4.21 (2.35–7.56)	3.31 (1.52–7.18)	3.08 (1.97–4.81)
**Negative likelihood ratio (LR-**) (**95 % CI**)	0.07 (0.01–0.46)	0.51 (0.30–0.87)	0.00 (0.00–0.31)
**Diagnostic odds ratio (DOR**) (**95 % CI**)	62.0 (7.3–530.2)	6.5 (1.9–22.0)	79.4 (4.4–1417.7)

## Discussion

This study demonstrated the importance of SQRP and DTP methods in diagnosing CLABSIs. The DTP method presented high specificity (82.5 %) reaching levels similar to those shown by Bouza *et al*. [14]. In practice, this means that a positive DTP result can prove that a BSI is central line-associated and therefore the CVC needs to be removed, apart from cases in which lock therapy is indicated [15]. The clinical utility of the latter can be optimized if using DTP combined with superficial cultures of the skin entry site, as displayed by previous studies [16]. In contrast, a previous study including a very low number of BSIs has revealed that the DTP method is not suitable for the diagnosis of CLABSIs in surgical critically ill patients [17].

SQRP displayed high sensitivity (94.7 %) and NPV (96.9 %) reaching percentages similar to previous studies [16]. This means that SQRP, which is commonly used by the majority of laboratories, can adequately preclude a CLABSI itself. The SQRP method presents the inability to culture bacteria from the internal lumen, however other techniques used to solve this problem have not shown any additional advantage [18]. However, the most important limitation of the SQRP method is that its performance requires prior CVC removal.

The combination of SQRP and DTP methods displayed 100 % sensitivity and NPV, a result that is also supported by Gowardman *et al*. [16]. Despite the fact that the quantitative method is considered a reference standard [8, 19], it is not implemented in the majority of hospitals. Thus, the combination of negative SQRP and DTP results could rule out CLABSIs, as demonstrated in our study. A limitation of our study was that it was conducted retrospectively and did not include all ICUs of our hospital. In conclusion, when the quantitative method is not available, attempts should be made so that SQRP and DTP methods are evaluated in combination.
